# A Rare Case of Neck Sarcomatoid Squamous Cell Carcinoma With Brain Metastases

**DOI:** 10.7759/cureus.26179

**Published:** 2022-06-21

**Authors:** Ipsit Shah, Abrahim N Razzak, Abhishek Janardan, Brandon Laing, Nathan T Zwagerman

**Affiliations:** 1 Medicine, Medical College of Wisconsin, Milwaukee, USA; 2 Internal Medicine, Medical College of Wisconsin, Milwaukee, USA; 3 Neurosurgery, Medical College of Wisconsin, Milwaukee, USA

**Keywords:** sarcomatoid, brain met, sarcomatoid carcinoma of head-neck region, head and neck squamous cell carcinoma (hnscc), squamous cell carcinoma (scc)

## Abstract

Squamous cell carcinoma (SCC) is the second most prominent form of skin malignancy. It occurs most frequently in older males with fair skin complexion that have extensive sun exposure most commonly in their childhood. The metastatic presentation of SCC is rare and is most common in the lung. In this paper, we present the unique case of a 73-year-old patient with sarcomatoid squamous cell carcinoma in their posterior neck that metastasized to the brain.

## Introduction

Cutaneous squamous cell carcinoma (cSCC) represents one of the most prominent types of skin cancer alongside basal cell carcinoma and melanoma [1]. Pathologically, ultraviolet (UV) radiation is well accepted as the primary driver of cutaneous neoplasms via its modulation of the p53 gene [2]. Histologically, squamous cell carcinoma (SCC) is characterized through the presence of hyperkeratosis alongside prominent acanthosis, thickening of the rete ridges, irregular nests, and sheets of keratinocytes invading the dermis [3]. SCC is the second most common form of skin cancer diagnosed within the United States, trailing only basal cell carcinoma [1]. SCC has been noted to most commonly appear in light-skinned males above the age of 50 in areas of the body with extended UV light exposure, the most common locations being the face, neck, bald scalp, and forearms [4]. Metastasis of squamous cell carcinoma in areas of high sun exposure is relatively uncommon, yet it can take place, especially amongst immunocompromised patients [4]. Prevention of squamous cell carcinoma involves counseling patients to use sunscreen and wear protective clothing. A skin biopsy is compulsory for the diagnosis of squamous cell carcinoma [5]. Following diagnosis, surgery is the predominant mode of treatment for squamous cell carcinoma with Mohs micrographic surgery being the preferred modality of treatment for squamous cell carcinoma of the head and neck [6]. Non-surgical methods to reduce the risk of squamous cell carcinoma progression can include photodynamic therapy, topical 5-fluorouracil, or imiquimod [7]. Here we present the case of a 73-year-old male with brain-metastasized sarcomatoid squamous cell carcinoma from the posterior neck. 

## Case presentation

The patient is a 73-year-old Caucasian male with a past medical history significant for gout, hypertension (HTN), hyperlipidemia (HLD), coronary artery disease (CAD), chronic kidney disease (CKD), and diverticulitis who presented to the clinic for evaluation of a posterior neck lesion. Physical exam showed a 9 mm lesion on the posterior side of his neck in November 2020. A week later, he underwent biopsy and surgical excision of this primary lesion. Pathology results of the specimen demonstrated squamous cell carcinoma with positive margins. 

In February 2022, the patient presented to his primary care physician with concerns of sinus infection, head complaints, and altered mentation. A complete blood count, comprehensive metabolic panel, thyroid-stimulating hormone reflex, SARS-CoV-2 IgG antibody, and vitamin D 25-hydroxy were ordered. All tests returned unremarkable except SAR-CoV-2 IgG, which was positive. The patient’s altered mental status was attributed to his COVID-19 infection at the time. However, 4 days later in February 2022, the patient’s mental status worsened, and he presented to the ED with headache, dizziness, confusion, and right arm numbness.

**Figure 1:**
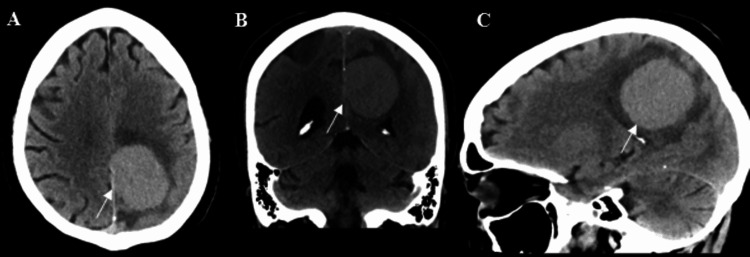
Initial CT Head imaging with axial (A), coronal (B), and sagittal (C) views. Imaging shows a hyperdense left frontoparietal mass along the falx cerebri with surrounding vasogenic edema.

**Figure 2:**
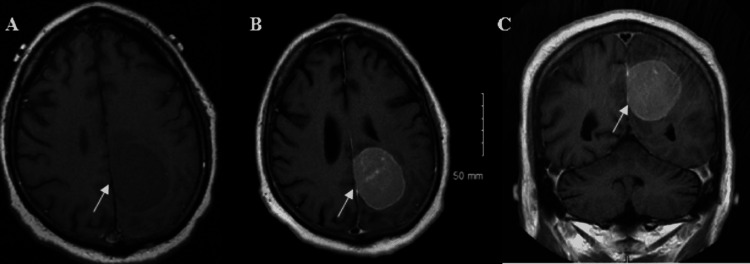
Initial MRI Brain with and without contrast. Noncontrasted T1 (A) and contrast-enhanced axial (B) and coronal (C) images show a homogeneously enhancing left frontoparietal mass.

A complete blood count, basic chemistry panel, ethanol, prothrombin time, partial thromboplastin, influenza/COVID-19 nucleic acid amplification test, microscopic and macroscopic urinalysis, and a urine drug of abuse screen reflex was ordered. Nothing significant was noted from any of the tests, except for the microscopic and macroscopic urinalysis which showed trace ketones and squamous epithelial cells. Given the negative laboratory work-up, a head computed tomography (CT) was ordered. The imaging showed a 4.9 cm lesion abutting the left posterior frontoparietal falx concerning for possible meningioma (Figure 1). Magnetic Resonance Imaging (MRI) of the brain was ordered to confirm the diagnosis. Imaging showed a contrast-enhancing left posterior frontoparietal mass (Figure 2).

**Figure 3:**
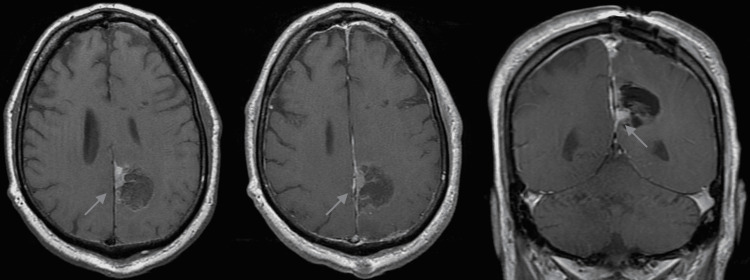
Postoperative non-contrasted (A) and contrast-enhanced axial (B) and coronal (C) images. Images show near-total resection with a small hyperintensity (blue arrow) within the inferomedial aspect of the resection cavity most consistent with blood products versus possible residual tumor.

Given the imaging findings, the patient was admitted to the Neurosurgery service for further work-up and pre-operative coordination. Two days later, the patient underwent a left craniotomy for tumor resection. Pathology was consistent for poorly differentiated sarcomatoid squamous cell carcinoma. Postoperatively, he successfully recovered from anesthesia, his neurological exam remained intact, and the left external ventricular drain was removed. The patient was transferred to the floor on postoperative day (POD) 1. A repeat CT and MRI showed no residual tumor and no postoperative complications (Figure 3). Given the pathology results, the patient underwent CT chest, abdomen, and pelvis as well as a lumbar puncture for cerebrospinal fluid (CSF) cytology. CT imaging did not show any other metastatic lesions. Cerebrospinal fluid was negative for infection or malignant cells. Postoperatively, the patient was discharged to an inpatient rehabilitation facility on POD 10. The patient was neurologically non-focal at the time of discharge

Three weeks postoperatively, the patient was treated with gamma knife radiosurgery to the resection cavity. Radiation was administered in three sessions with a total dose of 27 Gy. The patient tolerated radiation without complications.

One month after being admitted to the ED in March 2022, a positron emission tomography (PET) scan revealed that the cSCC had metastasized to the lung, several soft tissue sites, and most likely the lymph nodes. The patient was referred to palliative radiation therapy and prescribed pembrolizumab. He successfully completed a course of palliative radiation to the left lung to 25 Gy in 5 fractions. After radiation therapy, a subsequent MRI of his brain showed his cancer was stable. The patient is neurologically stable and is currently undergoing routine surveillance imaging.

## Discussion

Squamous cell carcinoma is the second most common skin cancer, behind basal cell carcinoma, with an approximate prevalence of 58.25 per 100,000 lightly pigmented individuals and 3.09 per 100,000 darkly pigmented individuals, oftentimes impacted by the exposure to UV light leading to malignant transformation of the cells [8]. The clinical presentation of SCC is extremely variable as it can oftentimes depend on location and subtype, however, head and neck squamous cell carcinoma (HNSCC) is a common SCC at 67% [8]. Besides UV light exposure, other risk factors include family history, genetic aberrations, and human papillomavirus (HPV). Many studies have evaluated the role of HPV in SCC pathogenesis due to the virus's effect on keratinocyte differentiation during its life cycle [9]. HNSCC is also known to have a high recurrence rate of primary lesions or distant metastasis, upwards to 50% within 5 years even with treatment of primary tumors [10]. The struggle in HNSCC control results from the robust lymphatic supply of the head and neck region. Due to the presence of close lymphatic nodes, HNSCC may present with local and distal metastasis post-diagnosis with lesions ranging anywhere from the ear to the oral cavity [11-13].

While HNSCC often spreads locally or to remote sites via the lymphatic system, it is rare for this pathology to metastasize to the brain. The most common brain metastases (BM) include primary tumors from lung cancer at 39% to 50%, breast cancer at 15% to 30%, and melanoma at 6% to 11% [14,15]. BMs are a rare sequela of HNSCC occurring in less than 1% of all reported cases with one multi-center review citing a rate of 0.3%. HNSCC BMs more commonly occur in males [16-18]. One hypothesis for this low rate of BM is that tumors of neuroepithelial origin such as the small cell carcinoma of the lung or melanoma can infiltrate the brain’s microenvironment at higher rates compared to epithelial origin cancer cells such as SCC where brain parenchyma finds it more difficult to transfer to [19,20]. While most reported cerebral metastases of SCC, as rare as it is, are HNSCC, there have also been select cases of SCC arising from distant areas such as the scrotum [21]. HPV positivity for HNSCC with BM has also been studied as a potential cause, however, in current reports, the incidence of BM in HNSCC is low at baseline, and reports of BM with HPV positivity are lacking [20]. Unfortunately, patients with BM do not have a great prognosis. One retrospective study involving 229 surgically treated BM patients counted mean survival time at 19.2 months, and patients with single metastasis at 17.6 months [22].

Here we present a rare case of poorly differentiated sarcomatoid squamous cell carcinoma (SSCC) that originated from the middle and outer layers of skin of the posterior neck and metastasized to the brain. The patient tolerated the operative left parietal craniotomy for resection of meningioma and the subsequent three gamma knife stereotactic radiosurgery sessions well for treatment. However, due to the metastatic nature of the disease, the patient has been referred to oncologic palliative radiotherapy for other lesions on his right neck, rib, and left lung.

Ultimately, the focus of the patient’s treatment as denoted above was through radiotherapy and pembrolizumab post-BM surgery. These measures were similar to chemo-radiotherapy and surgery treatment conducted in other examples of BM cases with thymic squamous and epithelial carcinoma [23,24]. There has also been discussion of using immune checkpoint inhibitors as a treatment for BM [25]. One of these is pembrolizumab, a recently FDA-approved treatment, used in this case of HNSCC with BM [26]. 

On top of the rarity of HNSCC with BM, it is also significant to notate that this patient case was diagnosed with SSCC, a rare subset of SCC. SSCC carries a rare morphological variant of sarcoma characteristics: cancer cells that affect connective tissue such as bones, blood vessels, and nerves [27]. SSCC frequently poses challenges in diagnostics, treatment, and management, whereas the underlying events for its evolution and progression from the conventional SCC continue to remain unknown [28]. Despite multimodality treatment, SSCC has been shown to act as a rare and aggressive disease variant with high degrees of mortality and recurrence rate [29]. One Taiwanese study reviewed 13,777 HNSCC cases from 30 years of hospital records and was only able to identify 78 cases of head and neck sarcomatoid squamous cell carcinoma (HNSSCC) [30]. While there have been cases reported in the past of SSCC with BM of pulmonary origin, this unique case of SSCC with BM is of head and neck origin [31-33]. 

Our case, while congruent to the poor prognosis that many BM patients have on top of the unique nature of HNSSCC, has stable brain MRI findings 5-months post-operatively, and the current focus of treatment is based on other metastasized sites such as the patient’s lung. The patient also carries comorbidities of gout, HTN, HLD, CAD, CKD, and diverticulitis netting a complex medication management course including allopurinol, bisoprolol fumarate, cephalexin, cetirizine, cholecalciferol, famotidine, gabapentin, omeprazole, prednisone, rosuvastatin, and terazosin alongside his chemotherapy. 

## Conclusions

This case of SCC with BM is unique due to its sarcomatoid characteristics alongside its head and neck origin of the primary tumor. Given the recent history, the patient was treated with recently FDA-approved immunotherapy, alongside the chemotherapy, gamma knife radiosurgery, and imaging surveillance protocols. While BM of HNSSCC is rare compared to metastasis from lung and breast cancer, early imaging studies of the brain may be considered for patients that present with neurological issues with a history of SSCC. 
